# An analysis of risk factors for visceral disseminated varicella in children

**DOI:** 10.3389/fped.2024.1345272

**Published:** 2024-05-30

**Authors:** Shuai Guo, Qin Guo, Chaomin Wan

**Affiliations:** ^1^ Department of Pediatrics, West China Second University Hospital of Sichuan University, Chengdu, China; ^2^ Key Laboratory of Birth Defects and Related Diseases of Women and Children, Ministry of Education, Chengdu, China; ^3^ National Health Commission Key Laboratory of Chronobiology, Sichuan University, Chengdu, China

**Keywords:** varicella, chickenpox, children, risk factors, severe infection

## Abstract

**Background:**

Visceral disseminated varicella involves the internal organs, and complications such as encephalitis, hepatitis, and coagulation disorders threaten a patient's life. In this study, our aim is to analyze the risk factors for visceral disseminated varicella to enable the early identification of patients at a high risk of visceral disseminated varicella.

**Methods:**

We reviewed the medical records of children hospitalized with varicella. The data covered demographics, clinical manifestations, auxiliary examinations, treatments, and outcomes. Logistic regression was used to analyze the risk factors.

**Results:**

A multivariate logistic regression analysis showed that abdominal pain [odds ratio (OR) 20.451, 95% CI 1.637–255.548], increased levels of C-reactive protein (OR 12.794, 95% CI 1.820–89.937), increased levels of alanine aminotransferase (OR 7.453, 95% CI 1.624–34.206), and the time between onset and antiviral therapy of more than 7 days (OR 12.451, 95% CI 1.569–98.810) were independent risk factors for visceral disseminated varicella.

**Conclusions:**

Patients with varicella who have the abovementioned risk factors need to be monitored for the risk of developing visceral disseminated varicella, for which timely antiviral therapy is necessary.

## 1 Introduction

Varicella (chickenpox) is a contagious disease caused by varicella-zoster virus infection, with the simultaneous existence of four types of skin lesions as its main clinical feature: macules, papules, herpes, and crusts. Varicella usually occurs in childhood, and is generally a benign, self-limiting disease that involves only the skin; some patients experience a fever and loss of appetite. Severe infections involve the internal organs, and complications such as encephalitis, hepatitis, and coagulation disorders threaten a patient's life.

Varicella is a vaccine-preventable disease. The first country to introduce universal varicella vaccination, the United States, managed to reduce the rate of incidence of varicella by 84.6% from the previous incidence rate after implementing a vaccination immunization strategy (1). In 1998, the World Health Organization recommended varicella vaccination in countries with a high public health burden. Many countries have achieved better results with the introduction of varicella vaccination (2). However, varicella remains an important worldwide public health problem because of its highly contagious nature and a large number of susceptible people. In 2014, the World Health Organization estimated that the annual global burden of varicella disease includes 4.2 million cases of severe complications that can lead to hospitalization and 4,200 deaths (3).

In this study, we aim to summarize the clinical characteristics of children with visceral disseminated varicella and analyze the risk factors for this disease condition to enable the early identification of patients at a high risk of severe infections.

## 2 Methods

### 2.1 Study population and setting

We reviewed the medical records of 75 children diagnosed with varicella between January 2012 and December 2021 at a tertiary medical center. The data covered demographics, clinical manifestations, auxiliary examinations, treatments, and outcomes.

### 2.2 Criteria of case selection

Varicella was diagnosed on the basis of clinical manifestations or laboratory criteria. Patients displaying a typical varicella rash provided clinical confirmation. Laboratory confirmation was provided by identifying the DNA of the varicella-zoster virus by polymerase chain reaction from a skin lesion or blood serum. Visceral disseminated varicella was defined by its presence in at least one internal organ of the patient.

### 2.3 Statistical analysis

All data were analyzed by using SPSS 25 statistical software. The measurement data of normal distribution were expressed as *X¯* ± *S*, and the Student’s *t*-test was used for comparison between patients belonging to two groups: the visceral disseminated varicella group and the non-visceral disseminated varicella group. The measurement data of non-normal distribution were expressed as M (Q1, Q3), and the comparison was performed by using the Wilcoxon rank sum test. Enumeration data were expressed as *N* (%). The categorical variable was tested by using the chi-square test. Logistic regression was used to analyze the risk factors. A value of *P* < 0.05 was considered statistically significant.

## 3 Results

### 3.1 Characteristics of patients with visceral disseminated varicella

**Figure 1:**
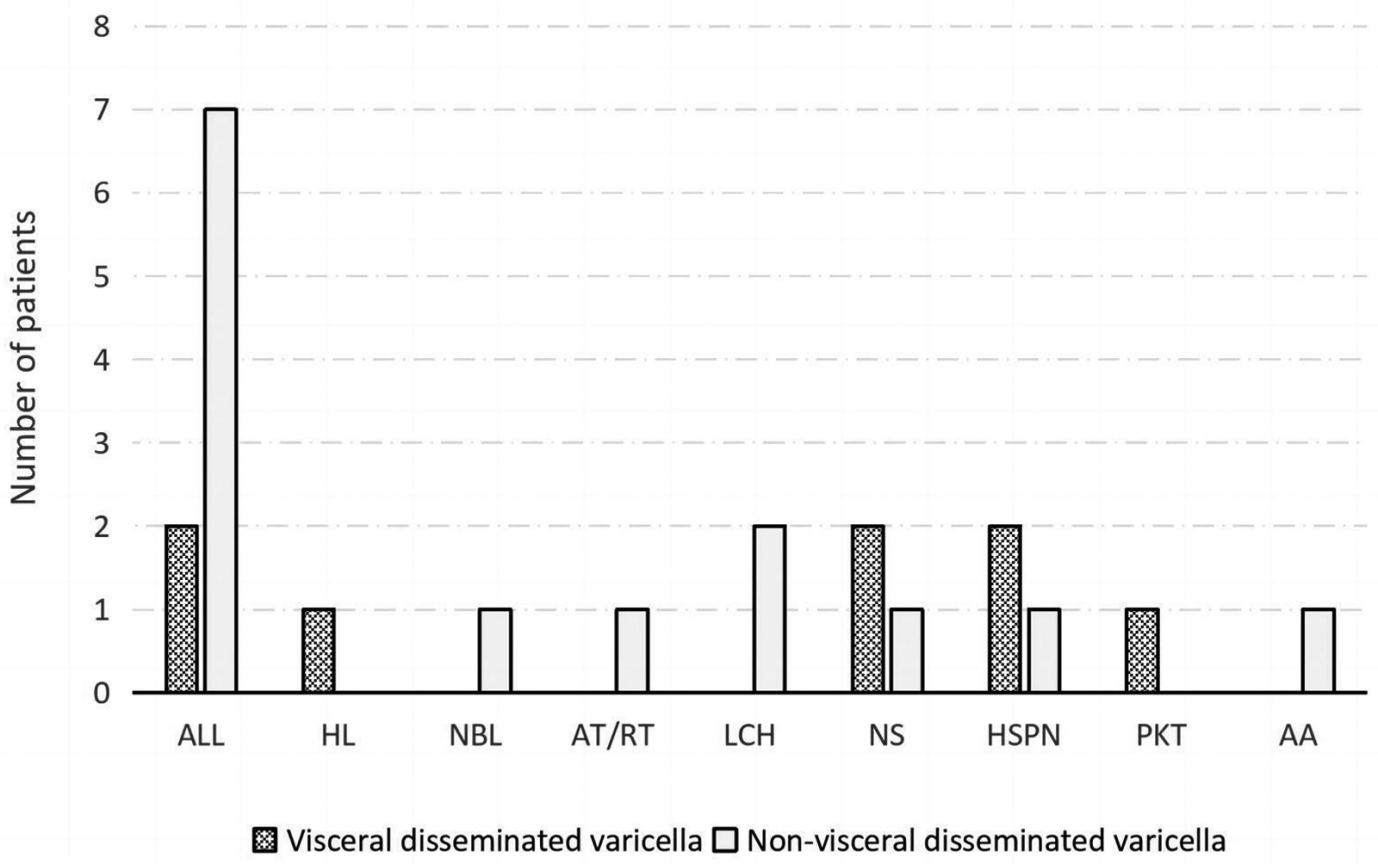
Comorbidities of patients belonging to the visceral disseminated varicella and non-visceral disseminated varicella groups. ALL, acute lymphoblastic leukemia; HL, Hodgkin lymphoma; NBL, neuroblastoma; AT/RT, atypical teratoid/rhabdoid tumor; LCH, Langerhans cell histiocytosis; NS, nephrotic syndrome; HSPN, Henoch–Schönlein purpura nephritis; PKT, post-kidney transplantation; AA, aplastic anemia.

The results demonstrated that 30.7% (23/75) of patients had visceral disseminated varicella. The age of onset was 7 (2, 11) years, in 13 male and 10 female patients. Of these, 78.3% (18/23) had received at least one dose of vaccine. Seven patients had a contact history, and eight had comorbidities (Figure 1). Five had been on low-dose corticosteroids for more than 1 month before the onset of the disease, and one had received chemotherapy within 21 days before onset. The remaining two patients had not received immunosuppressive therapy within the incubation period. The first symptom was a rash in 15 patients. Four had a delayed rash (more than 5 days between the first occurrence of any symptoms and the presence of a rash) and two had hemorrhagic herpes. Twenty-one patients had fever with a duration of 3 (1, 4) days. Eleven patients had two or more complications. The complications with the number of patients in brackets are given as follows: pneumonia (13), pneumorrhagia (3), respiratory failure (4), pleural effusion (2), encephalitis (6), encephalorrhagia (2), myocarditis (4), hepatitis (6), gastrointestinal hemorrhage (3), renal injury (2), sepsis (8), disseminated intravascular coagulation (7), and multiorgan dysfunction (4). Three patients died within 72 hours of admission because of cardiac arrest. The rest were cured and discharged within 11 (8, 17) days of hospitalization, and no sequelae were found on follow-up.

### 3.2 Univariate analysis

Data from the visceral disseminated varicella group and the non-visceral disseminated varicella group were analyzed by performing a univariate analysis, and the data pertained to demographics, clinical manifestations, auxiliary examinations, and treatments. The differences between the two groups were found to be statistically significant in terms of age (7 vs. 2.5 years, *P* = 0.023), abdominal pain (34.8% vs. 1.9%, *P* < 0.001), neutrophil count (4.2 × 10^9^/L vs. 2.3 × 10^9^/L, *P* = 0.003), increased C-reactive protein (CRP) levels (34.8% vs. 5.8%, *P* = 0.001), increased alanine aminotransferase (ALT) levels (65.2% vs. 28.8%, *P* = 0.003), use of corticosteroids (21.7% vs. 3.8%, *P* = 0.025), use of antibiotics (73.9% vs. 32.7%, *P* = 0.001), duration of antiviral therapy (7 vs. 6 days, *P* = 0.027), duration of hospitalization (11 vs. 7 days, *P* < 0.001), and the time between onset and antiviral therapy of more than 7 days (39.1% vs. 3.8%, *P* < 0.001) (Table 1).

**Table 1:** Clinical features and independent risk factors of visceral disseminated varicella.

Variable	Univariate analysis				Multivariate Logistic regression analysis		
	VDV (*n* = 23)	NVDV (*n* = 52)	Z/χ^2^	*P*	B	*P*	OR (95% CI)
Age (years)	7 (2, 11)	2.5 (0.5, 6)	−2.278	0.023			
Gender (female)	10 (43.5)	21 (40.4)	0.063	0.802			
Varicella vaccination	18 (78.3)	32 (61.5)	2.007	0.157			
Contact history	7 (30.4)	14 (26.9)	0.098	0.755			
Comorbidity	8 (34.8)	14 (26.9)	0.475	0.491			
Fever	21 (91.3)	46 (88.5)	0.135	0.713			
Duration of fever	3 (1, 4)	2 (1, 4)	−0.696	0.486			
Abdominal pain	8 (34.8)	1 (1.9)	16.305	<0.001	3.018	0.019	20.451 (1.637–255.548)
WBC (×10^9^/L)	8.3 (5.7, 14.1)	7.4 (6.0, 9.1)	−1.149	0.250			
Neutrophil count (×10^9^/L)	4.2 (2.8, 9.9)	2.3 (1.6, 4.2)	−2.965	0.003			
CRP>30 g/L	8 (34.8)	3 (5.8)	10.725	0.001	2.549	0.010	12.794 (1.820–89.937)
ALT>40 U/L	15 (65.2)	15 (28.8)	8.790	0.003	2.009	0.010	7.453 (1.624–34.206)
AST>40 U/L	15 (65.2)	34 (65.4)	<0.001	0.989			
The time between onset and antiviral therapy >7 days	9 (39.1)	2 (3.8)	15.863	<0.001	2.522	0.017	12.451 (1.569–98.810)
Duration of antiviral therapy	7 (6, 10)	6 (3, 7)	−2.215	0.027			
Use of corticosteroid	5 (21.7)	2 (3.8)	—	0.025			
Use of chemotherapy	3 (13.0)	11 (21.2)	0.691	0.406			
Use of antibiotics	17 (73.9)	17 (32.7)	10.934	0.001			
Duration of hospitalization	11 (8, 17)	7 (3, 8)	−3.951	<0.001			

VDV, visceral disseminated varicella; NVDV, non-visceral disseminated varicella; WBC, white blood cells; AST, aspartate aminotransferase.

### 3.3 Multivariate logistic regression analysis

The significant factors from the univariate analysis were analyzed by using a multivariate logistic regression analysis, and the results showed that abdominal pain [odds ratio (OR) 20.451, 95% CI 1.637–255.548], increased CRP (OR 12.794, 95% CI 1.820–89.937), increased ALT (OR 7.453, 95% CI 1.624–34.206), and the time between onset and antiviral therapy of more than 7 days (OR 12.451, 95% CI 1.569–98.810) were independent risk factors for visceral disseminated varicella (Table 1).

## 4 Discussion

Severe infection often occurs in immunocompromised patients. Previous studies compared the clinical features of varicella patients with primary and acquired immunodeficiency. Patients with primary immunodeficiency showed typical symptoms of varicella, including a rash, and the involvement of other organs, including the lungs and the central nervous system. Patients with acquired immunodeficiency (immunosuppressive treatment for nephrotic syndrome, solid organ transplantation, or hematological disorders, etc.) experienced a severe and fulminant course of the disease, with multiorgan failure, without or with delayed skin rash (4). In this study, patients with comorbidities had acquired immunodeficiencies because of receiving immunosuppressive therapy, especially visceral disseminated varicella patients with long-term corticosteroid use, a delayed rash, and in whom the disease worsened rapidly, following which two died. These two had been treated with high-dose corticosteroids and maintained on low-dose therapy during latent varicella; both of them had a very short course of the disease, characterized by circulation failure and disseminated intravascular coagulation. A study on the correlation between corticosteroid use and severe varicella showed an estimated 178-fold higher risk of severe varicella in children who had used corticosteroids within 30 days before the onset of varicella, compared with children who had not used them (5). Our study likewise found a correlation between corticosteroid use and the development of severe varicella.

In general, immunocompromised patients because of antineoplastic therapy are more susceptible to severe infections than immunocompetent patients. However, in this study, those receiving antineoplastic therapy patients are not at high risk of severe varicella. Probably because they saw a doctor and received antiviral treatment timely when they were unwell. We found that the time between onset and antiviral therapy of more than 7 days was an independent risk factor for visceral disseminated varicella; that is, the more the delay in starting antiviral therapy, the more likely the patient will develop visceral disseminated varicella. Another study also found that early and immediate antiviral treatment was effective in preventing a severe course of varicella (6).

Vaccination is an important measure to prevent varicella disease and its severe course. Currently licensed vaccines are either monovalent (varicella only) or combined with the measles, mumps, and rubella vaccine (MMRV). Countries and regions with varicella vaccination programs have used either one or two doses of vaccine, and, although there are different vaccination schedules, most studies with longer follow-ups saw reductions of more than 80% in disease incidence and hospitalization rates (2). A multicenter randomized controlled study showed that one dose of monovalent varicella vaccine was 65.4% effective against all varicella and 90.7% effective against moderate to severe varicella, while two doses of the combined vaccine were 94.9% effective against all varicella and 99.5% effective against moderate to severe varicella (7). Another study, which summarized 25 years of experience with varicella vaccine effectiveness research in the United States, found that a single dose of varicella vaccine provided moderate protection against any severity of varicella (82%–85%) and high protection against severe varicella (100%), but protection weakened over time (8). However, findings on whether vaccine-induced immunity and/or effectiveness wane after varicella vaccination are inconsistent (9–13). In China, varicella vaccination has not yet been included in the National Immunisation Programme, and is a voluntary vaccination, with the 1-dose varicella vaccination strategy used in most provinces and cities. In our study, although the vaccination rate was found to be high in the visceral disseminated varicella group of patients (78.3%), the older age of onset compared with the patients in the non-visceral disseminated varicella group may account for the reduced protective efficacy of the vaccine.

We recognize that there are limitations to our study. The sample was not big enough, we can't continue to develop a prediction model. It needs time to collect more cases to resolve. Although the global burden of varicella is lower than that of other major infectious diseases (such as measles, pertussis, mumps, or rotavirus), given that other vaccine-preventable diseases are better controlled, varicella may represent a considerable burden of preventable disease in the population, with significant healthcare and social costs. Therefore, it is a cause for concern.

In conclusion, our study found that abdominal pain, CRP >30 mg/L, ALT >40 U/L, and the time between onset and antiviral therapy of more than 7 days were independent risk factors for visceral disseminated varicella. Patients with varicella who have these risk factors need to be monitored for the risk of developing visceral disseminated varicella, for which timely antiviral therapy is necessary.

## Data Availability

The raw data supporting the conclusions of this article will be made available by the authors without undue reservation.
